# The effects of nebulized ketamine and intravenous magnesium sulfate on corticosteroid resistant asthma exacerbation; a randomized clinical trial

**DOI:** 10.1186/s40733-021-00081-1

**Published:** 2021-11-30

**Authors:** Kimia Farshadfar, Maryam Sohooli, Ramin Shekouhi, Ali Taherinya, Mostafa Qorbani, Mehdi Rezaei-kojani

**Affiliations:** 1 Alborz University of Medical Sciences, Karaj, Iran; 2 Colorectal Research Center, Department of Surgery, Shiraz University of Medical Sciences, Shiraz, Iran; 3 Department of Emergency Medicine, Shahid Rajaei Educational and Medical Center, Alborz University of Medical Sciences, Taleghani Boulevard, Taleghani Square, P.O. Box 31497-79453, Karaj, Iran; 4 Department of Epidemiology and Vital Statistics, Alborz University of Medical Sciences, Karaj, Iran

**Keywords:** Asthma, Ketamine, Peak expiratory flow rate, Magnesium sulfate

## Abstract

**Background and aims:**

Asthma exacerbation is defined as an acute attack of shortness of breath with more than 25% decrease in morning peak flow compared to the baseline on 2 consecutive days, which requires immediate standard therapy. The majority of asthmatic patients are considered to be steroid-sensitive; however, corticosteroid-resistant asthma is a subset of asthma with poor response to corticosteroids and is responsible for frequent hospital admissions. In this study we aimed to compare the effects of two enhancing strategies, the nebulized ketamine and IV magnesium sulfate, in treatment of severe steroid resistant asthma.

**Materials and methods:**

This double-blind randomized clinical trial was conducted on patients who presented to a referral clinic in Alborz, Iran. Using random allocation, patients were divided into two groups. The first group was treated with nebulized ketamine and the second group was treated with intravenous magnesium sulfate. Peak expiratory flow rates were assessed before the intervention, 30 and 60 min after the intervention and compared with the aid of SPSS software.

**Results:**

The Peak expiratory flow rates before the intervention, 30 min and 60 min after the intervention was statistically significantly different in both ketamine and magnesium sulfate groups. Peak expiratory flow rates change between 0 and 60 min were 29.4 and 15.2% in the ketamine and magnesium sulfate group respectively. Although the ketamine group showed much higher increase in mean PEFR compared to the MgSO_4_ groups, there was no statistically significant difference across both groups.

**Conclusion:**

Our study concluded that combined with standard therapy, both ketamine and IV magnesium sulfate are effective agents in the improvement of PEFR in patients with acute severe asthma that failed to respond to traditional therapies. However, there were no statistically significant difference between the two groups.

## Introduction

Asthma is a heterogenous chronic respiratory disease usually characterized as reversible airflow obstruction that presents with symptoms including shortness of breath, wheezing and cough caused by airway hyperresponsiveness to stimuli [1]. The pathophysiology of asthma involves an antigen-mediated inflammatory cascade causing immediate airway smooth muscle contraction, mucosal injury and edema, and ventilation perfusion mismatch [2]. Acute asthma exacerbation is account for most emergency department admissions and requires special attention [3]. Moreover, it is considered as one of the major causes of morbidity and mortality among asthmatic patients which is associated with life-threatening complications and variability in response to different therapies [4, 5]. Asthma exacerbation is defined as an acute attack of shortness of breath with more than 25% decrease in morning peak flow compared to the baseline on 2 consecutive days, which requires immediate standard therapy [6]. The initial asthmatic attack therapy comprises of oxygen supplementation, inhaled beta-2 agonists, and oral or parenteral corticosteroids. Fortunately, the prevalence of asthma exacerbation is declining as a result of well-developed preventive measures by national asthma guidelines [1].

The majority of asthmatic patients are considered to be steroid-sensitive, which is defined as achievable disease control with the aid of glucocorticoids and β_2_-adrenergic agonists. However, corticosteroid-resistant asthma is a subset of asthma with poor response to corticosteroids and is responsible for frequent hospital admissions in approximately 5–10% of asthmatic patients [7]. The exact pathophysiology of steroid-resistant (SR) asthma is not well understood. However, increased production of the IL-17A in pulmonary secretions seem to be associated with both severe and SR asthma. IL-17A, produced primarily by a distinct CD4+ TH cell subtype (TH17), is a proinflammatory cytokine that has been correlated with airway hyperresponsiveness and poor response to glucocorticoids [8–10].

For steroid-resistant asthma, second-line pharmacological therapies are currently being tested. They consist of steroid-sparing medications including magnesium sulfate, parenteral beta-2 agonist, intravenous (IV) aminophylline, or ketamine. However, there is no consensus regarding their therapeutic benefits and superiority. Ketamine for instance, is a sedative/analgesic agent mainly used for procedural anesthesia. However, its putative beneficial effects in bronchospasm management of SR asthma are less clear. Limited studies have shown promising results regarding its effects on bronchodilation and treatment of recurrent SR asthma due to its sympathomimetic properties, particularly in children [11]. Intravenous magnesium sulfate has emerged as another bronchodilator agent which reduce the rate of hospital admission amongst asthmatic patients. Its main mechanism of action modulating pulmonary bronchodilation is secondary to transient calcium channel blockage leading to smooth muscle relaxation and bronchodilation [12, 13]. However, the role of IV Mgso4 for refractory SR asthma remains relatively unexplored. In this study we aimed to compare the effects of two enhancing strategies, the nebulized ketamine and IV magnesium sulfate (Mgso4), in treatment of severe steroid resistant asthma.

## Materials and methods

### Study design

This randomized double blind clinical trial was conducted on patients who referred to emergency department of Shahid Rajaei Educational and Medical Center, Alborz, Iran from 2019 to 2020 with the chief complaint of severe asthma exacerbation*. Also, all patients who were aged between 18 and 65 years and patients who showed resistance to corticosteroids as a treatment for asthma exacerbation, were included in this study. Furthermore, patients who had the history of ketamine and sulfonamide sensitivity reaction in addition to the patients who did not consent were excluded from the study. The sample size was calculated to be 35, in which Zα =1.96, Zβ = 0.85, S = 62, d = 42 [14]:

$$\mathrm{n}=\kern0.5em \left[\left(\mathrm{Za}/2\kern0.5em +\kern0.5em \mathrm{Z}\upbeta \right)2\kern0.5em \times \kern0.5em \left\{2(6)2\right\}\right]/\left(\upmu 1\hbox{-} \upmu 2\right)2\Big]$$

*Severe asthma defined as failure to improve symptoms, blood oxygen saturation and persistent respiratory distress (peak expiratory flow < 50%) following first-line regimen administration. As we mentioned before, the first-line treatment options include IV hydration, oral/IV corticosteroids, nebulized beta-2 adrenergic (i.e., salbutamol) and muscarinic anticholinergics.

### Randomization and intervention

Patients were chosen to be in the ketamine and magnesium sulfate groups based on a 1:1 allocation using block randomization method and GraphPad Software. Both groups received primary treatments at first and patients in the ketamine group received nebulized ketamine (0.1–0.3 ml/kg); for patients in the magnesium sulfate group 2 g of MgSO_4_ was infused intravenously over a period of 20 min. Patients of both groups were assessed for PEFR 30 min and 60 min after the intervention by a peak flow meter (SIBEL, Spain) after a deep inhalation with the same measurement technique.

### Statistical analysis

Data were entered to version 25 of SPSS software for final analysis. Quantitative variables were reported as mean ± SD and qualitative variables were reported as numerical (percentage) data. The Anderson-Darling test was used for assessing the normality of data. Considering the normality of data based on the Anderson-Darling test, two-way repeated measures ANOVA was used for analyzing PEFR changes between two groups. Furthermore, independent t-test was used for measuring the differences of PEFR between two study groups.

## Results

Seventy patients were enrolled in this study with mean age of 40.9 ± 10.6. There were 36 (51.43%) male subjects and 34(48.60%) females, which were divided into two groups of ketamine and MgSO_4_. There were not any significant differences between groups in terms of gender distribution (*P* value = 0.8). The mean of age in ketamine and MgSO_4_ groups was 39.4 ± 9.7 and 41.9 ± 11.5 respectively. Also, there were not any significant differences in the context of age between two groups (*P* value = 0.4).

**Fig. 1:**
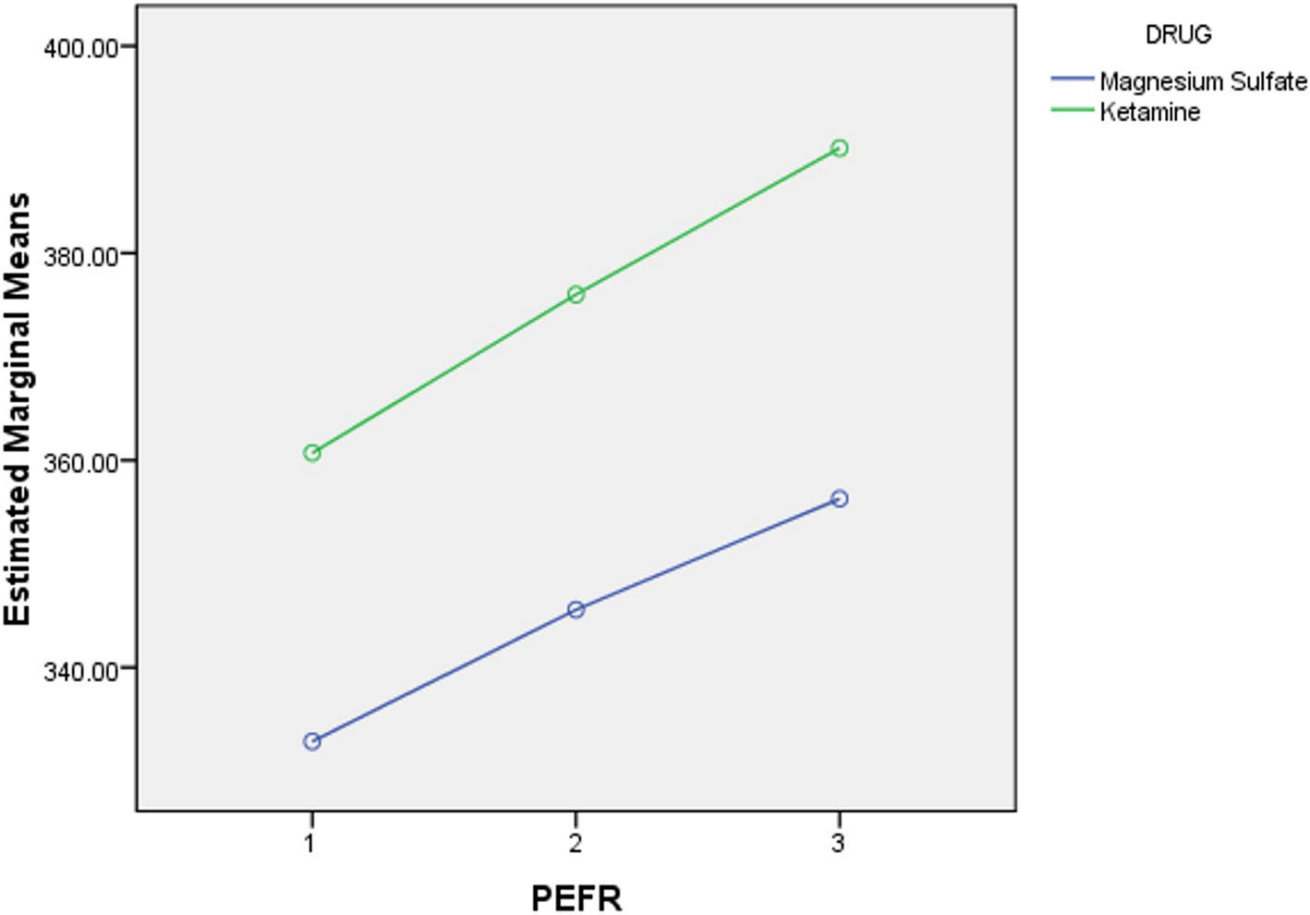
Comparing PEFR between the study groups

In the ketamine group, the rate of hospitalization was 46% (*n* = 13). According to Table 1, the mean of PEFR before the intervention was 360.71 ± 83.31. The mean of PEFR 30 and 60 min after ketamine administration increased to 376.0 ± 81.28, and 390.12 ± 79.44, respectively. As seen on Table 2, the mean PEFR after 60 min of drug administration had 29.42% increased. The observed finding in ketamine group in both males and females were statistically significant (*P*-value < 0.001) (Fig. 1).

**Table 1:** PEFR values in the study groups

PEFR	Value (mean ± SD)		*P* value*
	Ketamine group (n = 35)	MgSO_**4**_ group (***n*** = 35)	
Before the intervention	360.71 ± 83.31	332.85 ± 74.72	0.46
30 min after the intervention	376 ± 81.28	345.57 ± 71.8	0.32
**Mean Difference**	15.29 ***P-value*** **< 0.001**	12.71429 ***P-value*** **< 0.001**	
60 min after the intervention	390.14 ± 79.44	356.28 ± 71.98	0.15
**Mean Difference**	14.15 ***P-value*** **< 0.001**	10.71429 ***P-value*** **< 0.001**	

*Independent t-test

**Table 2:** PEFR changes in both groups

PEFR	Changes (%)		*P* value*
	Ketamine group (n = 35)	MgSO_4_ group (n = 35)	
Between baseline and 30 min	23.42%	12.71%	0.1
Between baseline and 60 min	29.42%	15.28%	
	***P-value*** **< 0.001**	***P-value*** **< 0.001**	

*Repeated measures ANOVA

**Fig. 2:**
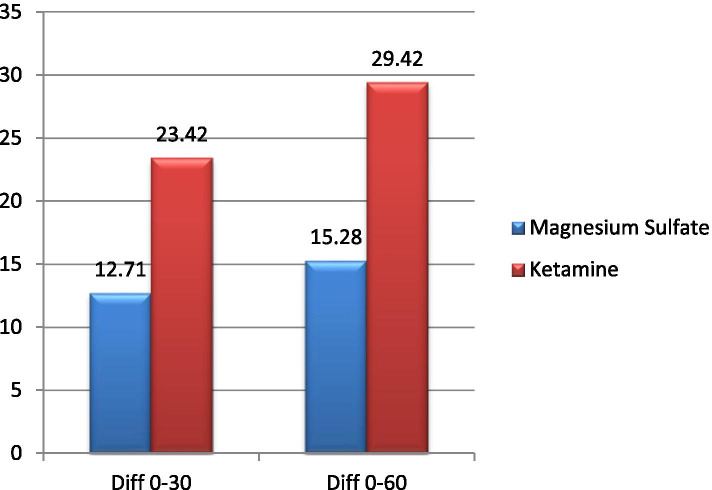
Comparing PEFR changes between the study groups

In the MgSO_4_ group, the rate of hospitalization was estimated to decreased to 54% (*n* = 15). Accordingly, both groups showed significant reduction in hospital admission. However, there were no statistically significant difference between two groups (ketamine and MgSO_4_) in terms of rate of hospitalization (P-value = 0.5). In the MgSO_4_ group, the mean value of PEFR in patients before the intervention was 332.85 ± 74.72. Moreover, the values of PEFR 30 min and 60 min after MgSO_4_ administration were 345.57 ± 71.80 and 356.28 ± 71.98, respectively (Table 1). Accordingly, the mean PEFR had increased 15.28% compared to baseline in the MgSO_4_ group (Fig. 1). These findings were also statistically significant (*P*-value < 0.001). Although the ketamine group showed much higher increase in mean PEFR compared to the MgSO_4_ groups, there was no statistically significant difference across both groups (*P* value = 0.1) (Fig. 2).

## Discussion

In the current study, the effects of ketamine and magnesium sulfate in the cases of steroid-resistant (SR) asthma were evaluated. This study reveals that nebulized ketamine can significantly improve PEFR in steroid resistant asthma exacerbation. Also, intravenous magnesium sulfate (MgSO_4_) showed promising results in treatment of severe SR asthma. However, the PEFR improvements were not significant between the two groups. To the best of our knowledge, this is the first study which compares the effects of nebulized ketamine and magnesium sulfate in the SR severe asthma exacerbation.

As we mentioned before, asthma is a heterogenous disease with various phenotypes caused by airway hyperresponsiveness resulting in inflammation, and expiratory airflow limitation that clinically presents with wheezing, cough, and dyspnea [1]. According to its inflammatory pattern, asthma has been classified into two groups of “T helper 2 (Th2)/ type 2 asthma” and “non-Th2/ type 2 asthma” [15]. The first group is consisted of exercised-induced, early-onset allergic, and late-onset eosinophilic asthma. Unlike early-onset allergic asthma which is considered steroid-responsive, the late-onset eosinophilic subtype is often resistant to corticosteroid. The main pathophysiology behind this steroid-resistance phenotype involves persistent sputum eosinophilia despite long-term use of corticosteroids [9]. The latter group, non-Th2/type 2 asthma, which includes obesity-related and neutrophilic asthma usually manifest with steroid-resistant airway inflammation. Airway neutrophilia and increased levels of cytokines release (particularly IL-17) have been suggested to be the cause of steroid-resistance [16, 17].

SR asthma has been reported to contribute to persistent airway inflammation, especially if left untreated [18]. Prolonged inflammation may lead to airway remodeling and permanent biomechanical alterations in airways [19]. Previous studies proven the fact that an imbalance between metalloproteinases and metalloproteinase inhibitors plays an important role in airway remodeling. These modifications include increased airway basement membrane thickening, angiogenesis, and smooth muscle mass [20, 21].

Generally, the first-line treatment of steroid-sensitive asthma is consisted of avoidance of allergen exposure, frequent use of bronchodilators, and glucocorticoids. However, SR phenotype of asthma should be suspected after lack of response following a 2-week course of oral/inhaled glucocorticoids administration. Thus, alternative treatment options are introduced when SR asthma is suspected in order to prevent the patients from on-going disease activity, and steroid adverse effects with lack of symptom relief.

Ketamine is a rapid-acting phencyclidine derivative with analgesic, sedative, and anti-emetic properties [22]. It can cause airway bronchodilation by interfering with various receptors and can interfere with inflammatory cascades which may result in bronchospasm modification [23]. We concluded that combined with standard therapy, ketamine is effective in the improvement of PEFR in patients with acute severe asthma that failed to respond to traditional therapies. Also, our study demonstrated that ketamine administration is generally effective in relieving bronchospasm and increasing blood oxygen saturation in SR asthmatic patients. Our study concluded that the mean PEFR raised significantly (*p*-value < 0.001) after 30, and 60 min of ketamine administration. Additionally, administration of the nebulized form of ketamine has no influence on central respiratory response compared to the intravenous form of ketamine. Thus, using the nebulized ketamine seems to be the safer treatment option, since it lacks the respiratory depression side effect of IV ketamine.

Accordingly, Betts et al. in 1971 demonstrated the effect of ketamine on asthmatic patient for the first time [24]. In the study of Petrillo et al., loading dose of 1 mg/kg ketamine followed by 0.75 mg/hour infusion showed significant relief in asthma symptoms [25]. Furthermore, in the study conducted by Esmailian et al., ketamine administration with the dosage of 0.4–0.5 mg/kg intravenously followed by infusion of the same dosage 30 min later, can rapidly increase the mean PEFR in patients with mild to moderate asthma [26].

Magnesium sulfate on the other hand, is a cellular hemostatic agent involved in histamine, and acetylcholine release leading to bronchodilation. It can also cause bronchial smooth muscle relaxation by interfering with calcium influx [27]. The current study demonstrated that IV MgSO_4_ probably provides desirable results in severe SR acute asthma in adult patients treated with conventional bronchodilators. In addition, we concluded that 2 g IV MgSO_4_ administration through 30 to 60 min, reduces hospital stay and improves PEFR significantly. Rowe et al., observed the similar results regarding the effects of IV MgSO_4_ as an adjuvant therapy [28]. Previous guidelines introduced IV MgSO4 as a safe and effective alternative treatment option for adult patients with acute severe SR asthma, who have not had sufficient response to first-line therapies [29]. However, the role of nebulized MgSO4 in treatment of severe SR asthma is not yet established. There have been few studies demonstrating the effects of nebulized MgSO4 in improvement of pulmonary function and decreasing the admission rate in asthmatic patient [30, 31]. Generally, use of nebulized MgSO4 is not fully recommended for treatment of severe SR asthma exacerbation in adults [29].

The last-line pharmacologic treatment for SR asthma includes cyclosporine, dapsone, gold salts, intravenous immunoglobulin (IVIG), and hydroxychloroquine. However, the majority of these medications are associated with significant risk of side effects and there are limited studies confirming their safety and efficacy. For instance, Alexander et al. [32] concluded that cyclosporine is associated with higher rate of morning PEFR and FEV1 with a 50% decreased chance of hospital admission in 33 patients with severe asthma. Methotrexate, an immunosuppressive agent with various anti-inflammatory properties, has been also studied for asthma management. However, most studies failed to show long-term remission and asthma control. Also, other pharmacological therapies such as IVIG have shown short-time pulmonary function improvement with lack of long-term beneficial effects. However, due to its high cost and adverse effects IVIG treatment is not generally recommended for severe asthma [33].

Overall, our study suggests that combined with standard therapy both ketamine and IV MgSO_4_ are highly effective in treatment of severe steroid-resistant asthma exacerbation in adults. Additionally, nebulized form of ketamine showed more desirable effects on improvement of respiratory function and decreasing hospital stay compared to IV MgSO_4._ However, these differences were not statistically significant.

A potential limitation of this study is the lack of interfering factors such as duration of asthma, number of previous attacks, severity of previous attacks and comorbidities in the study. It would be useful for future studies to consider these factors. Methodological limitations were small sample size, and short follow-up time. Also, another limitation of this study might be the fact that magnesium sulfate was administered as a standard dose of 2 g intravenously compared to ketamine, which was administered through a dose-dependent fashion with a nebulizer. However, it must be taken into consideration that the role of inhaled magnesium sulfate in management of severe steroid-resistant asthma is not generally recommended, which was the main reason for the use of IV MgSO_4_.

## Conclusion

Our study concluded that combined with standard therapy, both ketamine and IV magnesium sulfate are effective agents in the improvement of PEFR in patients with acute severe asthma that failed to respond to traditional therapies. Also, our study demonstrated that both therapies are generally effective in relieving bronchospasm and increasing blood oxygen saturation in severe steroid-resistant asthmatic patients. Additionally, nebulized form of ketamine showed more desirable effects on improvement of respiratory function and decreasing hospital stay compared to IV MgSO_4._ However, there were no statistically significant difference between the two groups.

## Data Availability

SPSS data of the participants can be requested from the authors. Please write to the corresponding author if you are interested in such data.

## Abbreviations

MgSO_4_: Magnesium sulfate
PEFR: Peak expiratory flow rate
SD: Standard deviation
IV: Intravenous
IVIG: Intravenous immunoglobulin
SR: Steroid resistanto
